# Superparamagnetic Iron Oxide Nanoparticles and Curcumin Equally Promote Neuronal Branching Morphogenesis in the Absence of Nerve Growth Factor in PC12 Cells

**DOI:** 10.3390/pharmaceutics14122692

**Published:** 2022-12-01

**Authors:** Mahshid Zarei, Abolghasem Esmaeili, Ali Zarrabi, Atefeh Zarepour

**Affiliations:** 1 Department of Cell and Molecular Biology & Microbiology, Faculty of Biological Science and Technology, University of Isfahan, Isfahan 8174673441, Iran; 2 Department of Biomedical Engineering, Faculty of Engineering and Natural Sciences, Istinye University, Istanbul 34396, Türkiye

**Keywords:** curcumin, superparamagnetic iron oxide nanoparticles, combination therapy, neuronal branching, neuroregeneration neuritogenesis

## Abstract

Regeneration of the damaged neurons in neurological disorders and returning their activities are two of the main purposes of neuromedicine. Combination use of specific nanoformulations with a therapeutic compound could be a good candidate for neuroregeneration applications. Accordingly, this research aims to utilize the combination of curcumin, as a neurogenesis agent, with dextran-coated superparamagnetic iron oxide nanoparticles (SPIONs) to evaluate their effects on PC12 cellsʹ neuronal branching morphogenesis in the absence of nerve growth factor. Therefore, the effects of each component alone and in combination form on the cytotoxicity, neurogenesis, and neural branching morphogenesis were evaluated using MTT assay, immunofluorescence staining, and inverted microscopy, respectively. Results confirmed the effectiveness of the biocompatible iron oxide nanoparticles (with a size of about 100 nm) in improving the percentage of neural branching (*p* < 0.01) in PC12 cells. In addition, the combination use of these nanoparticles with curcumin could enhance the effect of curcumin on neurogenesis (*p* < 0.01). These results suggest that SPIONs in combination with curcumin could act as an inducing factor on PC12 neurogenesis in the absence of nerve growth factor and could offer a novel therapeutic approach to the treatment of neurodegenerative diseases.

## 1. Introduction

One of the important issues related to neurodegenerative disorders is irreversible neuronal damage that can eventually lead to death [1,2]. It is estimated that more than 1.5 billion people worldwide suffer from central nervous system (CNS) disorders such as Alzheimer’s disease (AD), Parkinson’s disease, Huntington’s disorder, epilepsy, etc., which lead to an increasing number of deaths, especially in the elderly population [3,4,5,6,7]. These diseases are often immedicable, and the current treatments are only aimed at relieving or preventing progression of the symptoms in these patients [4,8]; therefore, the development of new, effective therapeutic approaches that could overcome the immedicably features of these diseases is an urgent need, and different research teams have focused on it [3].

Among neurotrophins such as brain-derived neurotrophic factor (BDNF), nerve growth factor (NGF), neurotrophin-4/5 (NT4/5), and neurotrophin-3 (NT3), nerve growth factor (NGF) plays a vital role in maintaining neuron growth, survival, and function [9,10]. Indeed, it could regenerate and reduce the process of neuronal degradation [10,11,12]; however, NGF is not a suitable candidate for the treatment of neurological diseases due to its short biological half-life and inability to cross through the blood–brain barrier (BBB) [12,13].

In this regard, natural compounds such as polyphenols and flavonoids could be an alternative therapeutic approach [10,14]. Over the last half-century, curcuminoids, especially curcumin, have been the subject of a wide range of biological and pharmaceutical research [15]. Curcumin is the main compound of the *Curcuma longa* plant and has a wide range of therapeutic properties, from antibacterial and anticancer activities to anti-inflammatory and neuroprotective effects [16,17,18,19]. In vivo and in vitro application of curcumin showed an enhancement in neurite outgrowth and proliferation [10,15,20]. Based on the results of research studies, curcumin is an ideal candidate for various neurological disorders [21,22,23]. Despite the remarkable drug activity of curcumin, low bioavailability resulting from its hydrophobic nature limits its therapeutic application [24,25,26,27]. Recently, different studies have been conducted in the field of neuroscience to improve the pharmacological performance of curcumin, among them is the application of nano-formulations [28,29,30,31]. Using a nano-drug delivery system is beneficial to improving the water solubility, bioavailability, and targeted delivery of curcumin [21,28,32]. Additionally, they have the capability of crossing the blood–brain barrier and providing a controlled release of drugs that could improve the bioavailability of curcumin in the brain, reduce its probable side effects on other cells, and achieve the desired dose in a specific time [33,34,35,36].

Most nanoparticles, such as SPIONs, have the potential to cross the blood–brain barrier and could be used as a targeted drug delivery system [35,36,37]. Little research has evaluated the effectiveness of iron oxide nanoparticles in neural regeneration, while the detailed mechanism of iron oxide nanoparticles in enabling neurogenesis is still not fully understood [1]. Moreover, platforms of iron oxide nanoparticles have magnetic properties and are used as contrast agents in magnetic resonance imaging (MRI) and the diagnosis of neurodegenerative disorders such as Alzheimer’s [38,39]. The results of many laboratory studies have shown that the functionalization of magnetic nanoparticles with biologically active molecules such as neurotransmitters and neurotrophic factors (NFs) during the differentiation process increases magnetic nanoparticles’ binding to the cells and could lead to more efficient results [40,41,42]. Kim et al. showed that simultaneous treatment of PC12 cells with NGF and iron oxide nanoparticles increased the number of neurite-bearing cells. It has also been revealed that their length prominently increased on 1, 3, and 5 days after differentiation induction [1]. The combination of bioactive molecules with nanoparticles (NPs) affects the activity and stability of molecules. Marcus et al. proved that the binding of nerve growth factor to iron oxide nanoparticles enhances the differential function of NGF in PC12 cells [43]. In our previous study, we proved that SPIONs could improve the efficiency of NGF and quercetin in terms of increasing the amount of neurite outgrowth in PC12 cells [40]. To the best of our knowledge, there is no study on the effectiveness of SPIONs, alone and in combination with curcumin, on neuronal outgrowth and branching morphogenesis in PC12 cells in the absence of nerve growth factor. Therefore, the current study aims to check the effect of SPIONs, curcumin, and Cur+SPIONs on the differentiation of PC12 cells in the absence of NGF. The cytotoxicity of curcumin and SPIONs on PC12 studied with MTT assay and the effects of SPIONs alone and in combination with curcumin on neurogenesis, and neural branching morphogenesis were evaluated using immunofluorescence staining and inverted microscopy.

## 2. Materials and Methods

### 2.1. Materials

Curcumin (diferuloylmethane), dimethyl sulfoxide (DMSO), poly-L-lysine hydrobromide, β3-tubulin monoclonal antibody, and FITC-conjugated secondary antibody, 3-(4,5-dimethythiazol-2-yl)-2,5-diphenyltetrazolium bromide (MTT), were purchased from Sigma-Aldrich Co., Ltd. (St. Louis, MO, USA); cresyl violet was prepared by Merck KGaA (Darmstadt, Germany), and coverslips were purchased from Marienfeld Co., Ltd. (Lauda-Konigshofen, Germany). NGF-β (GFM11) was bought from Cell Guidance Systems (St. Louis, MO, USA). RPMI medium, fetal bovine serum (FBS), trypsin, and penicillin–streptomycin were bought from the Bioidea Company (Tehran, Iran). Horse serum (HS) was purchased from Baharafshan Company. Dextran sulfate-coated superparamagnetic iron oxide nanoparticles (fluidMAG-DXS, article number 4105) were purchased from Chemicell GmbH (Berlin, Germany). PC12 cells were bought from the Pasteur Institute of Iran (Tehran, Iran).

### 2.2. SPIONs Characterization

The morphology features and size of the dextran sulfate-coated iron oxide nanoparticles were analyzed by scanning electron microscopy (SEM, Philips XL30: Eindhoven, The Netherlands), and the elemental composition of iron oxide nanoparticles was also evaluated using the energy dispersive X-ray spectroscopy (EDX) method. Here, powder of core-shell nanoparticles without coating was used for analysis. The hydrodynamic size and surface charge of the nanoparticles (dispersed in deionized water) were determined using Zeta-sizer (HORIBA SZ-100, Kyoto, Japan). Surface functional groups of the nanoparticles were determined using Fourier-transform infrared spectroscopy (FTIR, Jasco 6300, Tokyo, Japan). KBr tablet method was used for the measurement of the FTIR spectrum of nanoparticles between wavenumber of 400–4000 cm^−1^. Finally, the crystalline structure of the nanoparticles was evaluated by the X-ray powder diffraction (XRD, Bruker D8ADVANCE, Billerica, MA, USA) method between 2Ɵ = 15–90° using Cu Kα lamp.

### 2.3. Cytotoxicity

To determine the non-cytotoxic concentration of curcumin, PC12 cell viability was assessed by MTT assay. A quantity of 10 × 10^3^ PC12 cells was cultured in 96-well plates coated with poly-L-lysine and exposed to different concentrations of SPIONs, ranging from 10 to 100 µg/mL and 10–100 μM for curcumin for one, two, and three days. At the mentioned times cells were incubated with MTT solution (0.5 mg/mL) (Sigma Aldrich Co., St. Louis, MO, USA) for 4 h, and absorbance was measured by the ELISA reader at 560 nm (Thermo LabSystems Inc, Beverley, MA, USA). The MTT assay was performed according to the method of Mosmann, and absorbance was measured by the ELISA reader at 560 nm (Thermo LabSystems Inc, Beverley, MA, USA).

### 2.4. Immunocytochemistry

β3-tubulin marker was used as a neuromarker to confirm the stages of neural differentiation. A quantity of 1 × 10^5^ cells cultured in 12-well plates containing a coverslip was coated with poly-L-lysine and treated with NGF (50 ng/mL), SPIONs (20 µg/mL), curcumin (20 µM), and Cur+SPIONs; the concentration of curcumin was 20 µM in each Cur+SPION combination, and the concentration of SPIONs was also 20 µg/mL (each well containing 900 μL of SPIONs 22.22 µg/mL and 100 μL of curcumin 200 µM solved in low serum medium). In the next step, with the removal of culture medium from the cell surface, cells were fixed with 10% formalin for 20 min and were washed four to five times with PBS (1X). After the fixation step, cells were permeabilized with Triton (x-100) (%3) diluted in PBS and then were incubated with primary monoclonal antibody (β3-tubulin) (Sigma Co., St. Louis, MO, USA), diluted at 1: 500 in BSA (%1) blocking buffer for 1 h at 37 °C. Then the cells were washed four to five times with PBS (1x) and were incubated with secondary antibody (Sigma Co., St. Louis, MO, USA) diluted 1: 100 at BSA (%1) blocking buffer for 30 min at room temperature. The nuclei were labeled with DAPI for 15 min. The image of the stained cells was evaluated by light and fluorescent microscopy (fluorescent stereo microscope; Olympus Corporation, Olympus US SZX12, Tokyo, Japan).

### 2.5. Cresyl Violet Staining (Staining of Nissl Bodies)

The cresyl violet staining method was applied to study morphological differentiation in PC12 cells. A quantity of 1 × 10^5^ cells cultured in 12-well plates coated with poly-L-lysine and was exposed to NGF (50 ng/mL), SPIONs (20 µg/mL), curcumin (20 µM), and Cur+SPIONs; the concentration of curcumin was 20 µM in each Cur+SPION combination, and the concentration of SPIONs was also 20 µg/mL. After the removal of the culture medium, the cells were fixed with 10% formalin for 20 min, and the cells were then washed three times with PBS (1X). Finally, the cells were stained with 0.1% cresyl violet solution for 20 min, and the stained cells were washed three times with PBS (1X) for 5 min to remove excess dye. Cells were visualized and photographed with an inverted microscope (Olympus BX51, Tokyo, Japan) and evaluated for the presence of their bodies and neurites.

### 2.6. Neurite Outgrowth Analysis

A quantity of 1 × 10^5^ cells was seeded in a medium with high serum (5% FBS and 10% HS) in 12-well plates coated with poly-L-lysine; after one day, the medium with high serum was replaced with a low serum medium containing treatment. PC12 cells were treated with NGF (50 ng/mL), SPIONs (20 µg/mL), curcumin (20 µM), and Cur+SPIONs; the concentration of curcumin was 20 µM in each Cur+SPION combination, and the concentration of SPIONs was also 20 µg/mL (each well containing 900 μL of SPIONs 22.22 µg/mL and 100 μL of curcumin 200 µM solved in low serum medium). On the third and fifth days after treatment, by measurement of length, counting number of neurites, and neuronal branching, 3 different fields in each well with an average of 100 cells were evaluated using Image J software (NIH Image software version 1.43, Madison, WI, USA) according to the method presented by Kim et al. [1].

The level of differentiation was determined based on the rate of growth and increase in neurite length from L0 to L3. L0 refers to cells without neurites, L1 refers to cells whose length of neurites was shorter than the size of the soma, L2 refers to cells whose length of neurites was between the original size of the soma and twice the size of the soma, and L3 refers to cells whose length of neurites was longer than twice the size of the soma. Moreover, the number of neurites originating from soma was defined as N0 to N4 (N0 refers to a cell without neurites).

### 2.7. Statistical Analysis

Statistical analysis was performed using GraphPad Prism version 9.0 (GraphPad Software Inc., San Diego, CA, USA). The analysis method was a one-way ANOVA. A *p*-value < 0.05 was considered statistically significant. Data represent the mean ± SD from three independent experiments.

## 3. Results

### 3.1. SPIONs Characterization

**Figure 1:**
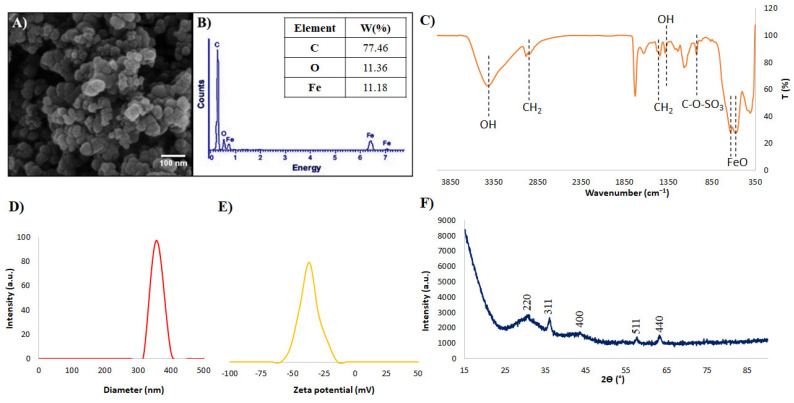
(**A**) SEM image, (**B**) EDX, (**C**) FTIR, (**D**) DLS, (**E**) Zeta, and (**F**) XRD analysis of superparamagnetic iron oxide nanoparticles coated with dextran sulfate.

In this research, SPIO-coated dextran sulfate nanoparticles with an iron oxide number of about 1.8 × 10^15^/g and a density of about 1.25 g/cm^3^ nanoparticles were used. The morphology, size, and elemental composition of the core–shell nanoparticles (NPs) were assessed by SEM and EDX analysis. According to Figure 1A, NPs have a spherical shape with a mean size of about 100 nm. Moreover, the presence of Fe, O, and C in the results of EDAX analysis confirmed the presence of both core and shell compounds in the structure of NPs (Figure 1B). According to the results of the FTIR test, fabrication of core-shell structure was confirmed due to the presence of Fe-O peaks of iron oxide core at around 550–600 cm^−1^, and hydroxyl (OH), alkyl (CH_2_), C–O–SO_3_, and groups of a shell at around 3400 and 1380, 2800–2900, and 1024 cm^−1^, respectively [44] (Figure 1C). Moreover, results of DLS and Zeta analyses display the presence of particles with a mean hydrodynamic diameter of about 300 nm and surface potential of about −37.1 mV (Figure 1D,E). High amounts of surface potential could confirm the stability of the nanoparticles; however, some aggregation was observed that led to an increase in the hydrodynamic size compared to the SEM result. In the XRD result, the existence of an amorphic peak at around 2Ɵ = 15°, which is related to the amorphous structure of dextran sulfate, and different peaks of iron oxide nanoparticles (including Miller indices of (220), (311), (400), (511), and (440)) that have accommodation with the crystallographic structure of the magnetite (JCPDS File no. 19-0629) [45].

### 3.2. Cytotoxicity

**Figure 2:**
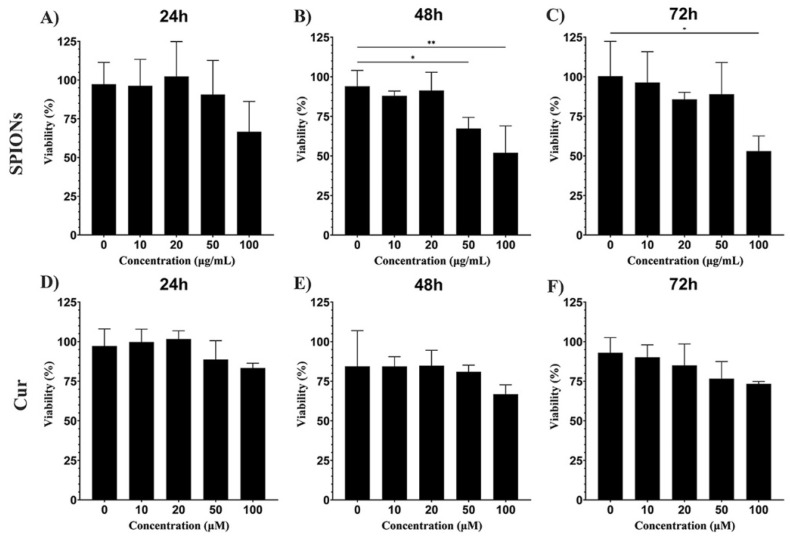
Effects of SPIONs and curcumin on the bioavailability of PC12 cells. (**A**) 24, (**B**) 48, and (**C**) 72 h after treatment of PC12 cells with 10, 20, 50, and 100 μg/mL. (**D**–**F**) PC12 cells were treated with 10, 20, 50, and 100 μg/mL curcumin for 24, 48, and 72 h. Data are presented as the mean ± SD, *n* = 3, * *p* < 0.05, and ** *p* < 0.01 vs. the control group.

To investigate the neurotoxicity of curcumin and SPIONs, the MTT assay was performed as explained in the Materials and Methods section. Results of the cytotoxicity test showed that increasing the concentration of SPIONs affected the viability of the cells, so a significant difference was observed compared to the control group (*p* < 0.01) (Figure 2A–C).

In the case of curcumin treatment, despite the decrease in viability at the concentration of 100 µM, no toxicity was observed at these concentrations. These data indicated that curcumin had no cytotoxic effect on PC12 cells (Figure 2D–F). Based on the results of the MTT assay and considering the results of the published articles [15,40], a dose of 20 μg/mL was selected for SPIONs and a dose of 20 μM for curcumin.

### 3.3. Immunocytochemistry

**Figure 3:**
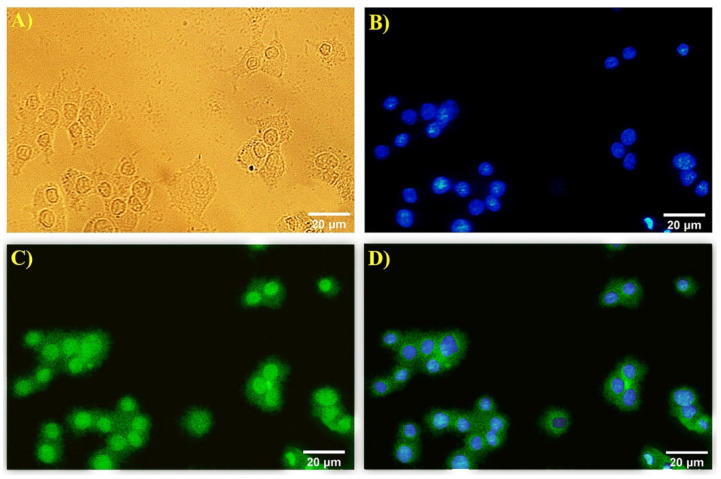
Immunofluorescence images of differentiated PC12 cells after 5 days of treatment with curcumin (20 µM). (**A**) Control, (**B**) DAPI staining, (**C**) β3-tubulin staining, and (**D**) the merge of (**B**) and (**C**). β3-tubulin fluorescently stained in green, and nuclei in blue.

Neurite outgrowth was studied in differentiated PC12 cells with anti-β3-tubulin by immunocytochemistry method. The expression of β3-tubulin was examined in cells treated with SPIONs, curcumin, and a combination of curcumin and SPIONs, and the expression of a neural marker protein related to neuritogenesis was observed in the PC12 cells in all treatment groups. Nuclei of cells are visualized in blue by DAPI and green areas indicate β3-tubulin expression in differentiated cells (Figure 3).

### 3.4. Neurite Outgrowth Analysis

**Figure 4:**
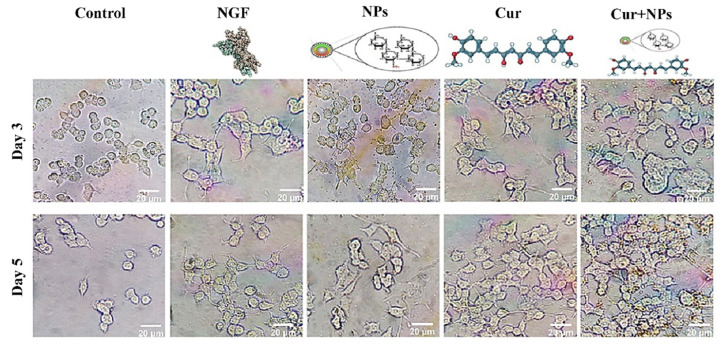
Typical images of differentiated PC12 cells. Microscopic images of differentiated PC12 cells 3 and 5 days after treatment with NGF (50 ng/mL), SPIONs (20 µg/mL), curcumin (Cur, 20 μM), and Cur+SPIONs (with a concentration of 20 µM for Cur, and the concentration of 20 µg/mL for SPION).

Investigation of the effect of curcumin and SPIONs and the combination of curcumin and SPIONs on neuronal differentiation was carried out in PC12 cells. Morphological differentiation (the length and number of neurites, and neuronal branching) was investigated for 3 to 5 days (Figure 4) after induction of differentiation in the control group and cells treated with NGF, SPIONs, curcumin, and Cur+SPIONs.

**Figure 5:**
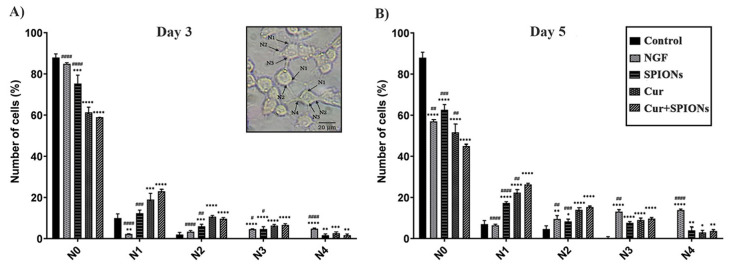
Effects of NGF, SPIONs, curcumin, and Cur+SPIONs on neurite outgrowth in PC12 cells. The number of neurites originating from the soma (**A**) 3 days and (**B**) 5 days after treatment with NGF (50 ng/mL), SPIONs (20 µg/mL), curcumin (20 μM), and Cur+SPIONs and the concentration of Cur was 20 µM in each combination Cur+SPIONs, and the concentration of SPIONs was also 20 µg/mL. Arrowhead indicates the number of neurites originating from the soma in cells treated with Cur+SPIONs. (#, * *p* < 0.05, ##, ** *p* < 0.01, and ###, ***, ####, **** *p* < 0.001).

The effectiveness of SPIONs on the differentiation function of curcumin was examined by evaluating the morphology of PC12 cells in the absence of NGF. The degree of differentiation was evaluated in terms of the number of neurites, neurite length, and neuronal branching. The effect of SPIONs on the neuritogenesis of curcumin was investigated at 3 to 5 days after being treated with NGF, SPIONs, curcumin, and Cur+SPIONs without adding nerve growth factor (Figure 5A,B). To quantitatively estimate the level of neuritogenesis, the number of neurites originating from soma was divided into five groups, N0 to N4, according to the method presented by Kim et al. [1]. The percentage of N0 cells (N0 refers to the cells without neurites) was examined and the same high percentage of differentiation in terms of neuritogenesis was observed in cells treated with Cur+SPIONs and curcumin without NGF compared to cells treated with NGF (*p* < 0.001), SPIONs (*p* < 0.001), and control (*p* < 0.001) 3 days after incubation with the mentioned treatments. The results of the data in Figure 5A imply that curcumin could increase equally the percentage of neurite-carrying cells without the effect of SPIONs and the absence of NGF in cells treated with curcumin and Cur+SPIONs at 3 days after induction of differentiation. Similarly, curcumin alone and without the influence of SPIONs was able to equally increase the percentage of N1, N2, and N3 cells (N1, N2, and N3 refer to the cells with 1, 2, and 3 neurites) in the cells treated with curcumin and Cur+SPIONs, and significant difference in terms of N1 (*p* < 0.001), N2 (*p* < 0.01), and N3 (*p* < 0.05) in the cells treated with SPIONs were observed at three days after incubation with the mentioned treatments. The percentage of N1, N2, N3, and N4 cells was studied in the cells treated with NGF, and despite the high percentage of N4 cells in NGF-treated cells, NGF cannot increase the percentage of N1, N2, and N3 cells and significant difference was observed in terms of N1 (*p* < 0.001), N2 (*p* < 0.001), and N3 (*p* < 0.05) between NGF-treated cells compared to treated cells with curcumin and Cur+SPIONs (Figure 5A).

The effectiveness of SPIONs on curcumin neuritogenesis without the presence of NGF at 5 days after incubation with the mentioned treatments also was investigated. Results shown in Figure 5B reveal a clear effect of SPIONs on the enhancement of the morphological function of curcumin and an increased percentage of the number of neurite-bearing cells in the absence of NGF when treated with Cur+SPIONs, in comparison with curcumin (*p* < 0.01), NGF (*p* < 0. 01), SPIONs (*p* < 0.001), and the control group (*p* < 0.001). The number of neurite-bearing cells in terms of N1 was also affected by SPIONs, and a high percentage of N1 was detected in cells treated with Cur+SPIONs in comparison to the cells treated with curcumin (*p* < 0. 01), SPIONs (*p* < 0.001), and NGF (*p* < 0.001). The data results in Figure 5B show that NGF enhanced neuritogenesis and increased the percentage of N3 and N4 cells in NGF-treated cells and were observed a significant difference in terms of N3 and N4 cells in cells treated with NGF (*p* < 0.01) alone and (*p* < 0.001) treatment compared to other groups, respectively. It should be noted that curcumin equally increased the percentage of N2 cells when compared to Cur+SPIONs and a significant difference was observed between cells treated with curcumin and Cur+SPIONs, and cells treated with SPIONs (*p* < 0.001) and NGF (*p* < 0.01). The results in Figure 5B show that SPIONs could improve neuritogenesis, and curcumin affected by SPIONs increases the percentage of neuritogenesis in terms of the number of neurite-bearing cells and N1 cells in cells treated with Cur+SPIONs without the presence of NGF after 5 days after incubation with the mentioned treatments.

SPIONs alone induced differentiation and increased the percentage of neurite-carrying cells (*p* < 0.001) on day 3 and the percentage of N1 (*p* < 0.001), N2 (*p* < 0.05), N3 (*p* < 0.001), and N4 (*p* < 0.01) cells on day 5 increased compared to the control group (Figure 5A,B).

**Figure 6:**
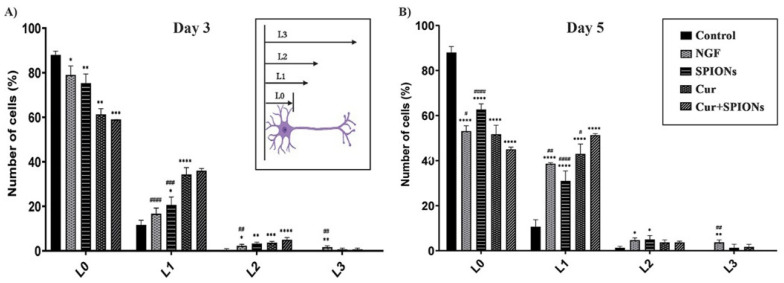
Distribution of the neurite length of PC12 cells at 3 to 5 days after treatment with NGF, SPIONs, curcumin, and Cur+SPIONs without the presence of nerve growth factor. (**A**) Evaluation of neurite outgrowth in terms of the length of the neurite at 3 days after induction differentiation with NGF (50 ng/mL), SPIONs (20 µg/mL), curcumin (20 μM), and Cur+SPIONs. (**B**) Effects of the mentioned treatments on the neurite length of PC12 cells at 5 days after induction of differentiation and the concentration of Cur was 20 µM in each combination Cur+SPIONs, and the concentration of SPIONs was also 20 µg/mL. (#, * *p* < 0.05, ##, ** *p* < 0.01, and ###, ***, ####, **** *p* < 0.001).

The length of neurites and neuronal branching without the presence of NGF were studied in PC12 cells treated with NGF, SPIONs, curcumin, and Cur+SPIONs. A high and equal percentage of cells with length L1 was observed with curcumin and Cur+SPIONs, and the data results show that curcumin could increase the percentage of L1 cells without being affected by SPIONs compared to cells treated with SPIONs (*p* < 0.001) and NGF (*p* < 0.001) at 3 days after treatment with curcumin and Cur+SPIONs (Figure 6A). Moreover, similar to the previous steps, the effect of SPIONs on the differentiation function of curcumin in terms of neurite length was investigated without NGF 5 days after being treated with NGF, SPIONs, curcumin, and Cur+SPIONs. The data results in Figure 6B show that neurite length was also affected by the SPIONs, and the enhancement of neurite length (L1) was observed in cells treated with Cur+SPIONs compared to cells treated with curcumin (*p* < 0.05), SPIONs (*p* < 0.001), and NGF (*p* < 0.01) 5 days after incubation with the mentioned treatments. In this research, we assessed the effect of SPIONs alone on neurite length. It was observed that SPIONs can increase the percentage of L1 (*p* < 0.05) and L2 (*p* < 0.01) cells within 3 days and L1 (*p* < 0.001) and L2 (*p* < 0.05) cells within 5 days after induction of differentiation compared to the control group (Figure 6A,B). Examining the length of neurites in cells treated with NGF for 3 to 5 days shows that NGF can increase the percentage of L3 cells (*p* < 0.01) compared to all treatment groups at 3–5 days after differentiation induction (Figure 6A,B). The data results of Figure 5B and Figure 6B indicate that SPIONs, by promoting the function of curcumin, could increase the percentage of N1 and L1 cells without NGF 5 days after incubation with Cur+SPIONs.

**Figure 7:**
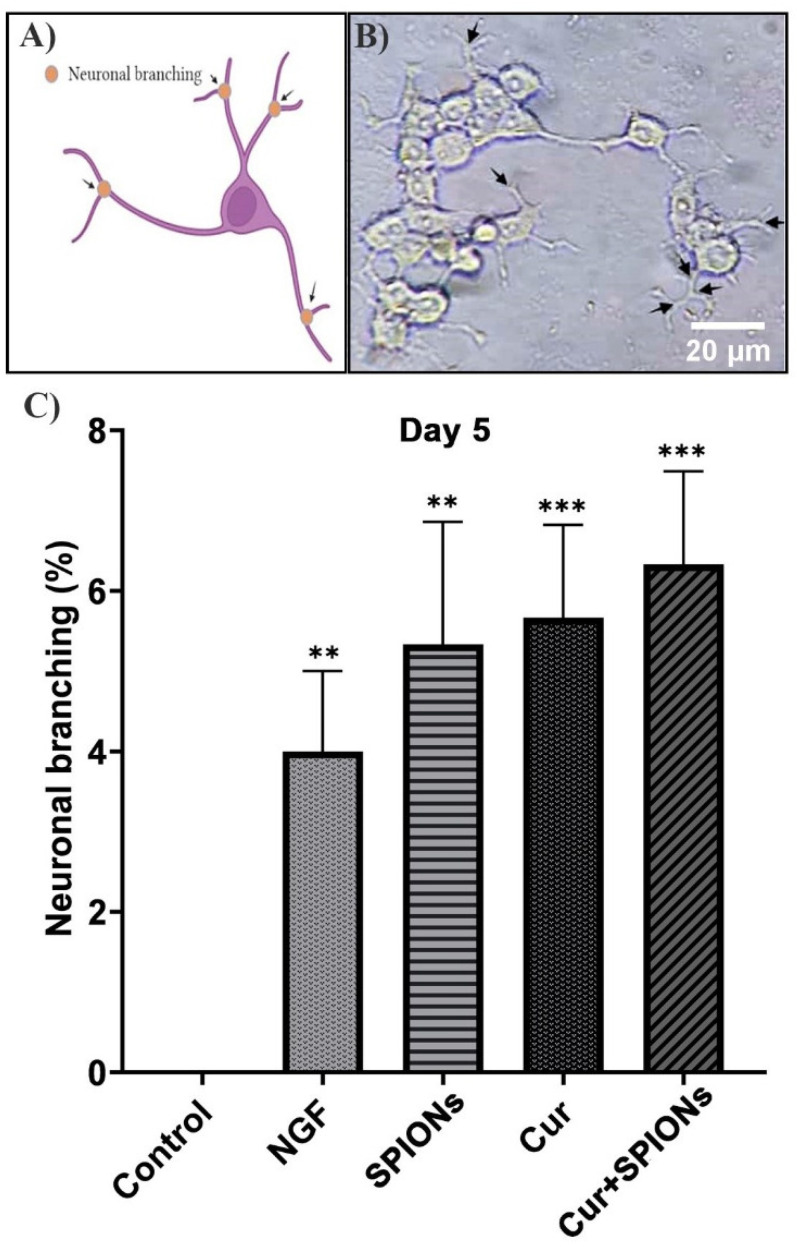
Evaluation of neuronal branching in differentiated PC12 cells. (**A**) Schematic image of assessment of neuronal branching. (**B**) Neuronal branching image in differentiated PC12 cells at 5 days after treatment with Cur+SPIONs and arrowhead indicates the site of neuronal branching in neurite-bearing cells. (**C**) Effects of NGF (50 ng/mL), SPIONs (20 µg/mL), curcumin (20 μM), and Cur+SPIONs on neuronal branching at 5 days after exposure to differentiation conditions at and the concentration of Cur was 20 µM in each combination Cur+SPIONs, and the concentration of SPIONs was also 20 µg/mL (** *p* < 0.01 and *** *p* < 0.001).

Like the previous parameters, the effect of SPIONs on morphological differentiation properties in terms of neural branching in each cell of neurite-bearing was measured in all treated cells 3 to 5 days after incubation with the mentioned treatments in the absence of growth factor according to Figure 7A. SPIONs are capable of inducing differentiation in PC12 cells and a clear morphological and identical effect on neuronal branching was observed in all treated cells with Cur+SPIONs (Figure 7B), curcumin, SPIONs, and NGF. SPIONs alone could increase the percentage of neuronal branching similar to curcumin (*p* < 0.001) and NGF (*p* < 0.01) 5 days after incubation with the mentioned treatments in PC12 cells (Figure 7C).

## 4. Discussion

The study of agents that promote the repair and recovery of lost neurons in neurodegenerative diseases is of great importance [43]. In this research, we examined the effect of SPIONs on the function of curcumin in the absence of NGF during neuronal differentiation of PC12 cells. Various investigations have been performed on bioactive compounds from natural resources with neurotrophic function as an alternative therapeutic approach; however, their therapeutic effects on neuronal differentiation in neurodegenerative diseases still need to be clarified [3,10]. Abundant research has been conducted on the medicinal effects of curcumin and it has been proposed as a potent neuroprotective compound for the remedy of age-related diseases such as Alzheimer’s disease [10,17,22,41,42,46]. However, the clinical application of curcumin is restricted by low aqueous solubility, poor stability, and a high rate of metabolism when applied both in vitro and in vivo [24,28,39,47].

We investigated, for the first time, the effect of SPIONs with curcumin on the differentiation morphology of PC12 cells in the absence of nerve growth factor. Various research has been conducted on combinations of nanoparticles with therapeutic pharmaceutical agents to overcome their limitations [21,25,28,48,49]. Marcus et al. demonstrated that functionalized iron oxide nanoparticles with NGF significantly promoted neuritogenesis in PC12 cells [43].

Before starting to evaluate Cur+SPION combination activity, we assessed the toxicity of curcumin and SPIONs. Cytotoxic effects of SPIONs and curcumin have been previously offered at high NPs doses. Our cell viability assays showed that at the concentrations measured, curcumin had no cytotoxic effect on PC12 cells, and when the concentration of SPIONs was less than 50 μg/mL, there was no significant difference in PC12 viability. The results of other investigations in the field of cytotoxicity of SPIONs and curcumin confirm our results. For instance, Fan et al. showed that PC12 cells treated with curcumin in the concentration range of 0–100 µM had no cytotoxicity. Liu et al. showed that SPIONs had dose-dependent cytotoxicity at 60–200 µg/mL [50,51].

To confirm neural morphogenesis in differentiated PC12 cells, an immunocytochemistry technique with anti-β3-tubulin was applied, and high expression levels of neuronal differentiation marker β3-tubulin, associated with neurite growth, were seen in treated cells in the absence of nerve growth factor. Moreover, the presence of β3-tubulin in differentiated PC12 cells with the compound of epigallocatechin gallate (EGCG) and curcumin with the immunofluorescence staining technique was investigated and approved by Dikmen et al. [10].

In this research, we described that SPIONs can improve curcumin function and promotion in the percentage of N1 and L1 cells 5 days after being treated with Cur+SPIONs in the absence of NGF. Studies have shown that drug loading onto iron oxide nanocarriers could improve these drugs’ therapeutic effects [21,43,48,49]. Nano formulation of curcumin improves curcumin’s pharmacological function in vivo and in vitro [28]. Curcumin-conjugated magnetic nanoparticles were colocalized with amyloid plaques in Alzheimer’s disease mice model and could have the potential for detecting amyloid plaques for diagnosis of Alzheimer’s disease using MRI [38]. The enhancement of NGF activity with a nanotechnology-based approach to the induction of differentiation and neurites outgrowth in PC12 cells was confirmed and evaluated by Marcus et al. [43]. Kim et al. demonstrated that non-conjugated NPs with NGF absorbed by PC12 cells could modulate the expression of adhesion molecules and enhance neurite outgrowth through the activating of the mitogen-activated protein kinase pathway (MAPK) [1]. The results of our research and other studies show that combination therapy can improve the potential of therapeutic agents such as curcumin.

To investigate the effectiveness of SPIONs alone on the differentiation activity of curcumin, cells were incubated with curcumin and a curcumin–SPION combination in the absence of NGF. We demonstrated that curcumin was not affected by iron oxide nanoparticles 3 days after induction differentiation, and can enhance the percentage of cells N1, N2, N3, and L1 in the absence of NGF, when it is alone. The studies conducted in this field also confirm the clinical potential of curcumin in the treatment of neurodegenerative diseases [52,53].

The finding of some reports has approved the ability of the neuroprotective effects of bioactive molecules, such as natural phenolic compounds, to reduce cell inflammation associated with neurodegenerative diseases through the elimination of reactive oxygen species (ROS) and disrupting existing amyloid plaques in several experimental models of Alzheimer’s disease [3,53,54,55,56]. In addition to anti-amyloid, antioxidant, neuroprotective, and anti-inflammatory effects, curcumin could induce differentiation and promote neurite outgrowth neurite in PC12 cells through the activation of dependent pathways MAPK/ERK and PKC in PC12 cells [15]. In a research study, Dikmen et al. showed that the combination of epigallocatechin gallate (EGCG) and curcumin synergistically could induce neuronal differentiation in PC12 cells [10]. Therapeutic potential and neuroprotective effect of curcumin against hippocampal damage with 6-hydroxydopamine (6-OHDA) in Parkinson’s rat model with increased expression of brain-derived neurotrophic factor (BDNF) and phosphatidyl receptor proteins inositol 3-kinase (PI3K), was evaluated and validated by Yang et al. [57]. Delivery of this therapeutic agent using poly(lactic-co-glycolic acid) (PLGA) nanoparticles could enhance neurogenesis by inducing the proliferation and differentiation of neurons via affecting different genes related to the Wnt/β-catenin pathway [47,58].

In recent years, different methods have been introduced for neuroregeneration applications, some among them utilizing nanomaterials [59,60]. In this research, we evaluated the effect of SPIONs on the differentiation of the PC12 cells without NGF, and we observed that SPIONs alone could increase the percentage of neuronal branching, similar to curcumin. The results of some research have shown that metal ions such as Mn and Fe could affect neuronal differentiation, among metal ions, Mn can enhance neural outgrowth with NGF and the absence of the nerve growth factor [1,61,62,63,64]. The application of magnetic iron oxide nanoparticles (MIONs) in combination with an external magnetic field can induce neural differentiation in mouse embryonic stem cells (mESCs) [65]. Additionally, the incorporation of these nanoparticles inside the PLGA nanofibers could enhance the expression of neuronal markers (such as neuron-specific class III beta-tubulin (Tuj 1) and neuron-specific enolase (NSE)) and induce differentiation of stem cells [66]. Different hypotheses have been proposed for the enhancement of the neural outgrowth by a metal ion, including that released Fe ions produced from iron oxide nanoparticles by organelles into the cell cytosol due to acidic pH inside the organelles could enhance neurite outgrowth [1,62]. The other hypothesis is that ROS induced by metal ions such as iron oxide nanoparticles could promote neurite initiation and elongation [67]. Some researchers have suggested ROS created by iron oxide nanoparticles, through the activation of the Akt pathway and with the ability to inhibit glycogen synthase kinase-3β (GSK3β), leads to a decrease in the phosphorylation of its target protein, microtubule-associated protein (MAP2), and causes changes in cellular actin dynamics and increases the length of neurites [68,69]. The results of our research and other studies show that nanoparticles, such as the therapeutic agents of nerve growth factor and curcumin and could induce differentiation morphology in PC12 cells.

NGF is essential for neuronal protection and outgrowth of neurons and also presents a high therapeutic potential for the treatment of Alzheimer’s and other neurodegenerative diseases [9,12,43]. Surprisingly, Higuchi et al. demonstrated that neuronal branching in NGF-treated PC12 cells suppresses PI3K-Akt signaling, whereas PI3K-Rac promoted neuronal branching [70].

We have shown that SPIONs can promote neuronal differentiation in combination with curcumin in the absence of nerve growth factor in PC 12 cells. Considering all these factors, using SPIONs as a nano-based approach improves the activity of therapeutic agents such as curcumin in the lack of NGF, and curcumin affected by SPIONs can enhance neurogenesis without nerve growth factor. The neuroprotective activity of the combination use of curcumin and SPION was also shown in a study conducted by Naserzadeh and her coworkers. They showed that curcumin could prevent the toxicity effects of SPION by reducing the production of ROS related to the presence of SPION; however, they didn’t have any study on the neuroregeneration effects of the combined use of these two compounds [71]. In other words, we propose the theory that the combined use of these two compounds could maintain the production level of ROS in their beneficial amounts for neuronal regeneration. Meanwhile, more studies are needed to confirm this hypothesis.

We also proved that nanoparticles, the same as nerve growth factor and curcumin, could induce differentiation in PC12 cells and increase nerve branching without the presence of nerve growth factor. It should be noted that to achieve an effective treatment method and use nanoparticles as drug carriers to improve the performance of bioactive molecules and eliminate side effects, points such as cellular absorption and intracellular fate of nanoparticles should also be considered [72,73]. In addition, using an external magnetic field can also play an effective role in active targeting and efficient treatment [43,74,75].

## 5. Conclusions

To summarize, combination therapy is an efficient method of improving the performance of therapeutic agents, and we have shown that a combination of curcumin with SPIONs increases the curcumin function for inducing neuritogenesis in the absence of nerve growth factor. Improving the neuritogenic activity of curcumin and overcoming its application limitations can increase its therapeutic effects in clinical fields. This approach can serve as an efficient method for improving the therapeutic potential of curcumin in the treatment of neurodegenerative disorders such as Alzheimer’s disease (AD).

## Data Availability

Not applicable.
